# Ehlers-Danlos syndrome and the risk of spontaneous colonic perforation: clinical insights and surgical implications

**DOI:** 10.1093/jscr/rjaf558

**Published:** 2025-07-25

**Authors:** Daniela Gaspar, João L Pinheiro, Andreia Santos, Carolina Canhoto, Rosa Simão, Raquel Barros Pereira, Jorge Pereira

**Affiliations:** General Surgery Department, Unidade Local de Saúde – Viseu Dão Lafões, Av. Rei Dom Duarte, 3504-009 Viseu, Portugal; Clinical Academic Center of Beiras (CACB), Edifício UBImedical, Estrada Municipal 506, 6200-284 Covilhã, Portugal; General Surgery Department, Unidade Local de Saúde – Viseu Dão Lafões, Av. Rei Dom Duarte, 3504-009 Viseu, Portugal; Clinical Academic Center of Beiras (CACB), Edifício UBImedical, Estrada Municipal 506, 6200-284 Covilhã, Portugal; Faculty of Health Sciences, University of Beira Interior, Convento de Santo António, 6201-001, Covilhã, Portugal; General Surgery Department, Unidade Local de Saúde – Viseu Dão Lafões, Av. Rei Dom Duarte, 3504-009 Viseu, Portugal; Clinical Academic Center of Beiras (CACB), Edifício UBImedical, Estrada Municipal 506, 6200-284 Covilhã, Portugal; General Surgery Department, Unidade Local de Saúde – Viseu Dão Lafões, Av. Rei Dom Duarte, 3504-009 Viseu, Portugal; Clinical Academic Center of Beiras (CACB), Edifício UBImedical, Estrada Municipal 506, 6200-284 Covilhã, Portugal; General Surgery Department, Unidade Local de Saúde – Viseu Dão Lafões, Av. Rei Dom Duarte, 3504-009 Viseu, Portugal; Clinical Academic Center of Beiras (CACB), Edifício UBImedical, Estrada Municipal 506, 6200-284 Covilhã, Portugal; General Surgery Department, Unidade Local de Saúde – Viseu Dão Lafões, Av. Rei Dom Duarte, 3504-009 Viseu, Portugal; Clinical Academic Center of Beiras (CACB), Edifício UBImedical, Estrada Municipal 506, 6200-284 Covilhã, Portugal; General Surgery Department, Unidade Local de Saúde – Viseu Dão Lafões, Av. Rei Dom Duarte, 3504-009 Viseu, Portugal; Clinical Academic Center of Beiras (CACB), Edifício UBImedical, Estrada Municipal 506, 6200-284 Covilhã, Portugal

**Keywords:** spontaneous colonic perforation, Ehlers-Danlos, autosomal dominant, case report

## Abstract

Ehlers-Danlos Syndromes (EDS) are a group of genetic connective tissue disorders classified into to 13 subtypes according to different genetic mutations. The vascular subtype, also known as Type IV, is considered the most severe subtype and is associated with dire complications. Spontaneous gastrointestinal perforation is the most commonly described digestive complication, with colonic perforation accounting for most cases. We report a challenging case of EDS, diagnosed with colonic perforation as the initial presentation of the disease.

## Introduction

Ehlers-Danlos syndrome (EDS) is a rare autosomal dominant connective tissue disease. EDS comprise a group of connective tissue disorder with 13 recognized subtypes [1]. The vascular subtype, vascular EDS (vEDS) or Type IV EDS, accounts for <4% of EDS cases and is considered the most severe form of presentation [1] [2]. Patients with vEDS have an increased risk for life-threatening complications at an early age, leading to a reduced life expectancy, with a mean age of survival of 50 years [1] [3]. Spontaneous gastrointestinal perforation is the most frequently observed digestive complication in these patients, with colonic perforation being the most commonly affected bowel segment [2].

This report describes the challenges faced in the diagnosis and surgical management of a young patient with spontaneous colonic perforation due to an undiagnosed EDS.

## Case report

The patient was a 25-year-old Caucasian male with a medical history of attention deficit hyperactivity disorder, managed with methylphenidate, non-traumatic repetitive shoulder dislocations, and smoking habits. Notably, his family medical history revealed that his mother had died, at a young age, from an unclear acute gastrointestinal incident.

**Figure 1:**
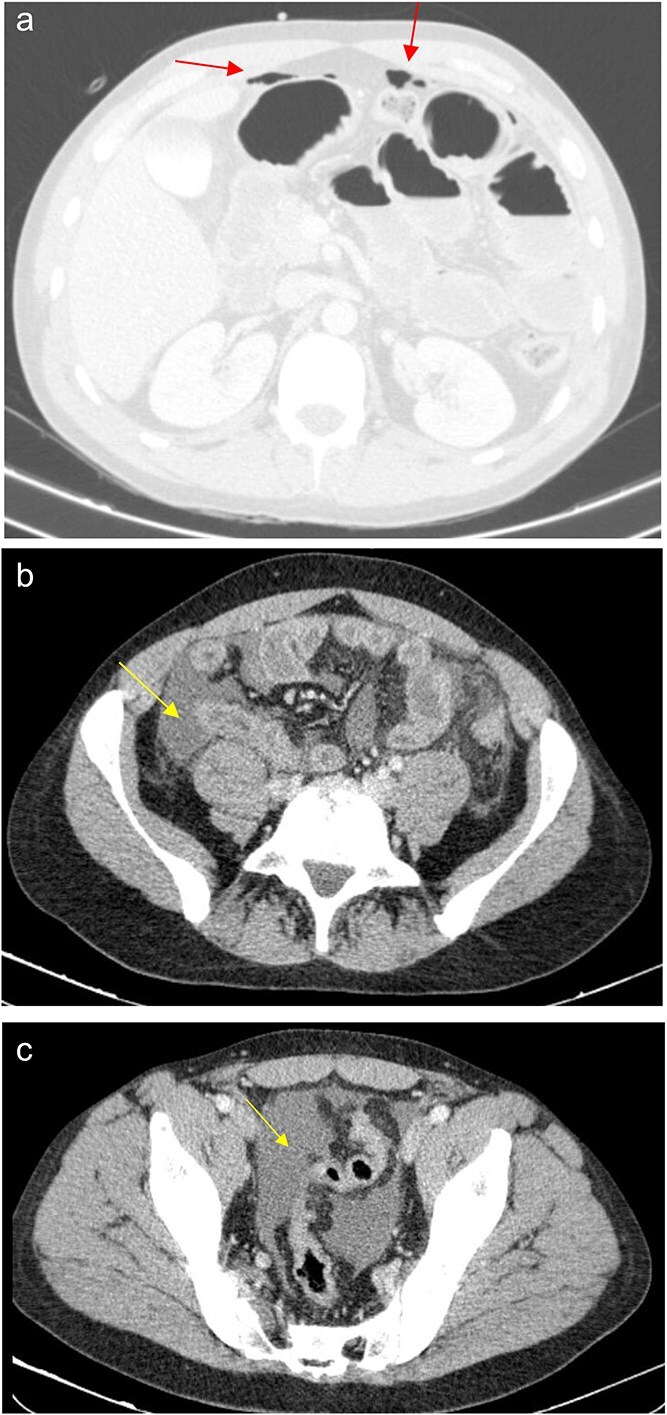
(a) Pneumoperitoneum (arrowheads). (b) Free fluid in the right iliac fossa (arrowhead). (c) Free fluid in the pelvis (arrowhead).

**Figure 2:**
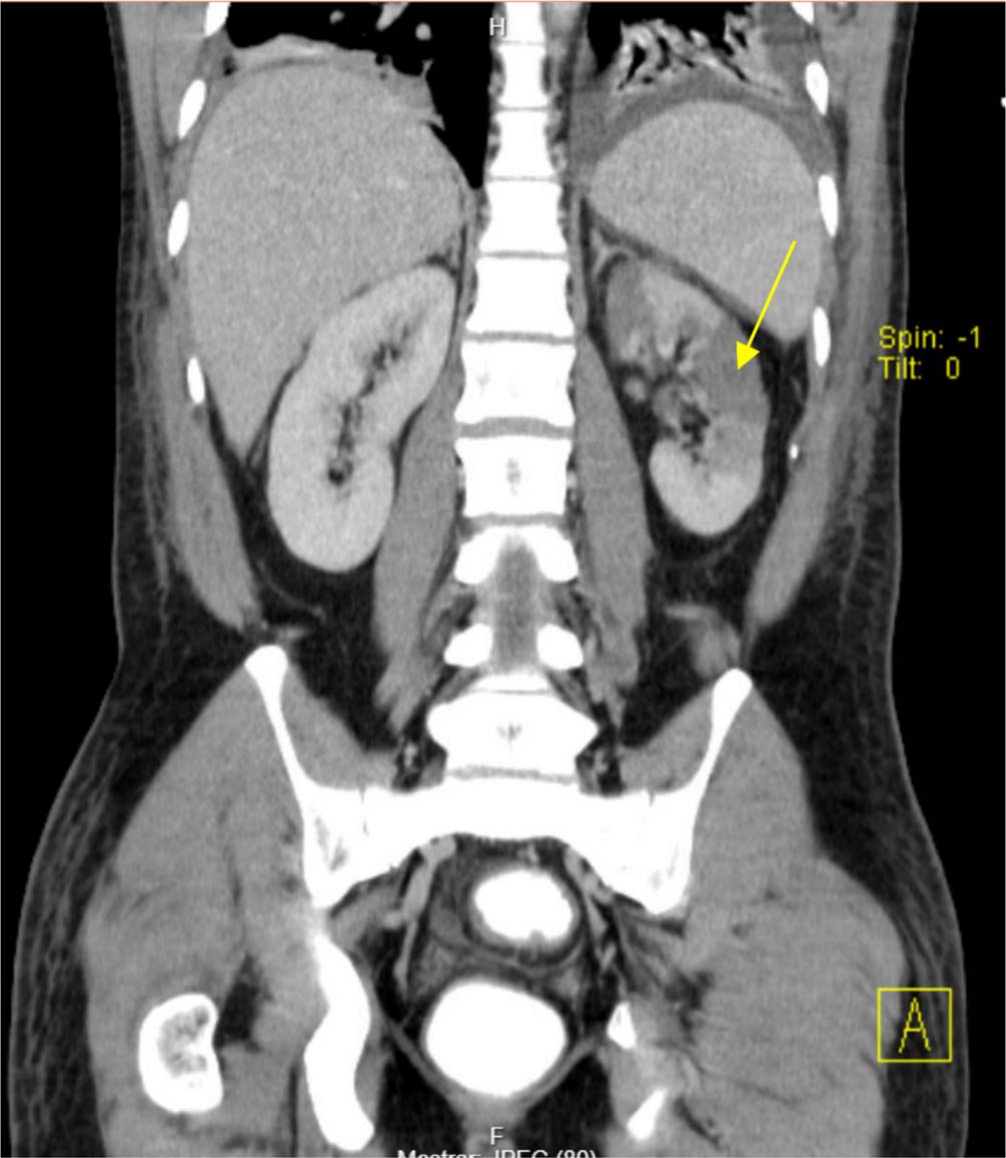
Left kidney infarction (arrow).

**Figure 3:**
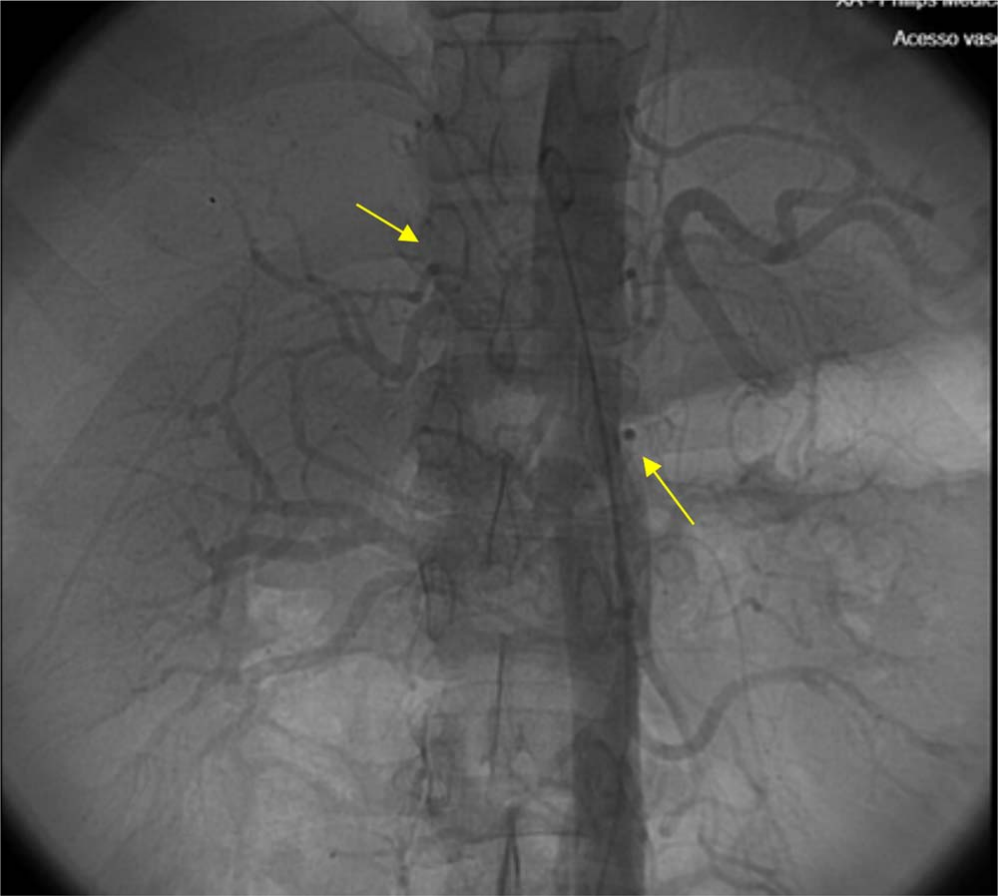
Angiography of the abdominal aorta revealed arterial microaneurysms suggesting polyarteritis nodosa (arrows).

**Figure 4:**
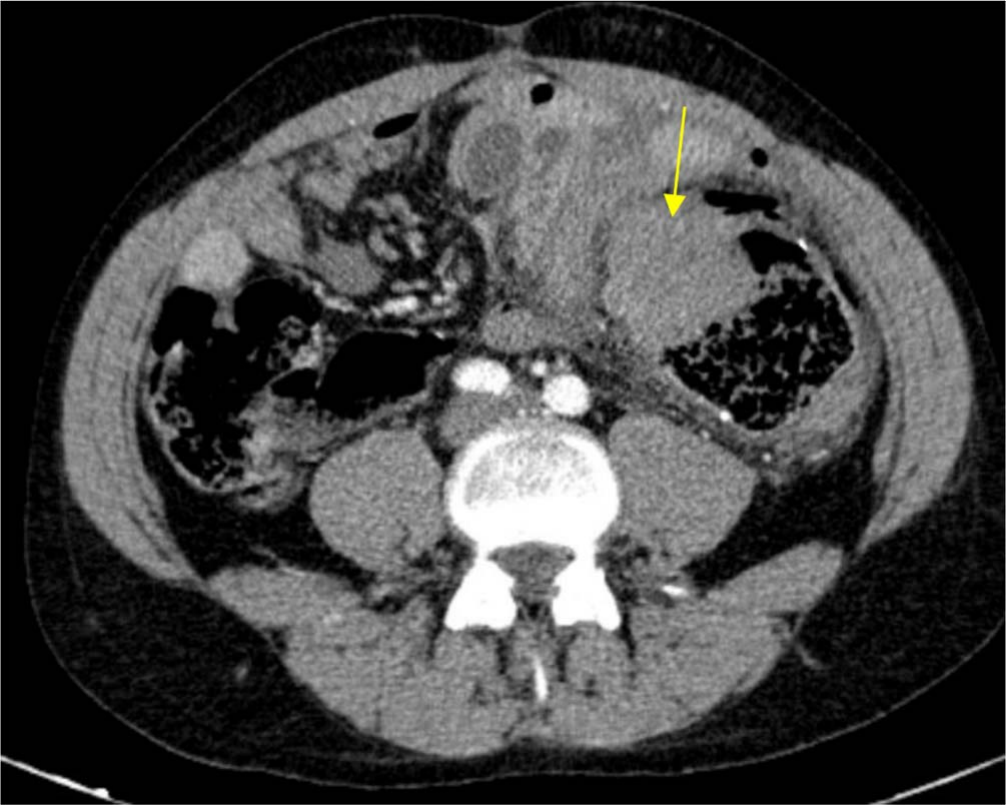
Circular densification of the sigmoid colon wall, distal to the previous anastomosis (arrow).

The patient presented at our Emergency Department with a sudden, diffuse, intense abdominal pain. Upon physical examination, the patient’s blood pressure was measured at 132/76 mmHg, heart rate of 103 bpm and a temperature of 39.4°C. The abdominal examination showed a rigid abdomen, with diffuse tenderness, suggesting an acute abdomen scenario. Laboratory tests revealed elevated inflammatory parameters. A abdominal and pelvic computed tomography scan revealed pneumoperitoneum and free fluid in the right iliac fossa and pelvis (Fig. 1). An exploratory laparotomy was proposed and the intraoperative findings included a sigmoid colon perforation with faecal peritonitis. They proceed with sigmoidectomy and temporary abdominal closure, planning for a reintervention to restore gastrointestinal continuity. This was done, after 48 h, through mechanical latero-lateral colo-colic anastomosis. The histopathological examination confirmed diverticulosis with perforation. The patient was subsequently transferred to the intensive care unit, for 5 days. Posteriorly, he developed severe left lumbar pain and hypertension unresponsive to medication. A CT scan revealed left kidney acute ischemia due to left renal artery thrombosis (Fig. 2). Broad-spectrum antibiotic therapy and anticoagulation, was initiated, after assessment by the Vascular Surgery and Urology teams. During his hospitalization, the patient experienced additional thrombotic events, such as occlusion of the left radial artery, and right lower lobar pulmonary embolism. A workup for prothrombotic conditions was conducted, including tests for thrombophilia, lupus, cardiolipins, antinuclear antibody, total protein test, and immunoglobulins, all of which were negative. An angiography of the abdominal aorta revealed arterial microaneurysms in the splanchnic territories: hepatic, perigastric and mesenteric arteries, suggesting polyarteritis nodosa (Fig. 3). Echocardiogram showed no abnormalities. The patient also underwent a study of the left shoulder due to repetitive pain complaints, with a suspected rupture of the rotator cuff. Electromyography excluded peripheral nerve damage. No other complications were reported. The patient was discharged on the 21st day after surgery, under anticoagulation therapy and steroids due to a suspected diagnosis of polyarteritis nodosa. Twenty-seven days after surgical intervention, the patient returned to the ED, presenting with abdominal pain in the lower quadrants and nausea. He was hemodynamically stable with abdominal pain and tenderness on the left flank and iliac fossa. Laboratory tests revealed leukocytosis and hyperlactacidemia. The CT scan reported free fluid, free gas bubbles adjacent to the sigmoid which had a concentric circular thickening of the wall, just distal to the previous anastomosis (Fig. 4). An exploratory laparotomy was performed, during which a large hematoma on the wall of the left colon was identified, with no apparent signs of perforation. A peritoneal lavage, drainage, and a derivative ileostomy were carried out. During the post-operative period the patient maintained a small volume of purulent discharge through the abdominal drain, with no abdominal tenderness. A reevaluation CT scan was performed, highlighting a regression of the hematoma of the colonic wall but raised suspicion of a low output fistula near the anastomosis. Since the patient maintained hemodynamic and analytical stability, he was discharged from the hospital with home care, including an abdominal drain, parenteral nutrition, empirical antibiotic therapy, and gradual reduction in the steroid dosage. The patient was referred to the ED, on Day 6, due to enteric drainage. An enterocutaneous fistula was diagnosed, and he was readmitted. He received 12 days of full-dose parenteral nutrition, which reduced the fistula output and improved his clinical and analytical status. Due to residual drainage volume and an analytical and imagiologic improvement, the drain was removed, and the patient was discharged, tolerating an oral diet, and with a functioning ileostomy.

A multidisciplinary approach was used to plan the patient’s treatment. During the follow-up outpatient appointments:

- Neurology: A contrast-enhanced magnetic ressonance imaging (MRI) revealed nonspecific leukoencephalopathy which was classified as possible leukodystrophy. This finding led to a genetic study that detected a mutation in the COL3A1 gene, indicating EDS.
- Orthopedics: An MRI of the left shoulder showed signs of a Hill-Sachs lesion related to the shoulder luxation.
- Internal Medicine: A positron emission tomography scan showed no signs of vasculitis. The patient ceased anticoagulation and began a gradual reduction of steroids.
- Immuno-hemotherapy: Thrombophilia tests (antithrombin III, and Proteins C and S) and Prothrombin studies showed no abnormalities.
- General Surgery: The patient remained asymptomatic.

## Discussion

vEDS is characterized by qualitative and quantitative abnormalities in type III collagen synthesis, a significant component of the blood vessel walls, skin, joint capsules, uterus and gastrointestinal tract, particularly the colon. This disorder is associated with mutations in the COL3A1 gene (rarely COL1A1), located on chromosome 2 at the q24.3 - q31 locus (OMIM 130050). This gene codes for the pro-α [1] chain of type III collagen and the transmission is of autosomal dominant inherence. However, the mutation rate is high (50%), which explains the occurrence of sporadic cases [3].

Spontaneous colonic perforations in young adults are rare due to the infrequency of colorectal tumors, complicated diverticular disease, or ischemic events in this age group [4]. However, intestinal perforation is characteristic of vEDS, and, in line with the presented case, some EDS diagnoses have occurred following bowel perforation. Most perforations happen in the sigmoid colon, occasionally in the small intestine. Approximately 31% of the vEDS patients experience recurrent colonic perforations, and complications like impaired wound healing, and anastomotic leak [5]. Currently, there is no established treatment for intestinal perforation in vEDS. Minimizing surgical stress is crucial as it can elevate blood pressure, increase collagenase activity, and lead to arterial complications. Some experts suggest that total colectomy with ileostomy may prevent recurrent perforations. However, the prognosis after such treatment remains poor. Prolonged postoperative monitoring using non-invasive imaging techniques is essential, and genetic counseling should be provided to the patient and their family members [1, 5, 6].

Screening and preventive measures for intestinal perforations in patients with vEDS remain unclear. An international group of specialists has developed recommendations to minimize injury in vEDS patients, including maintaining normal blood pressure and ensuring that patients carry medical information about their condition [5].

In this case, multidisciplinary management involving surgeons, physicians, and genetic counseling was required. Centralizing care at specialized centers is beneficial in order to provide the expert monitoring that such complex cases require.

## Conclusion

Spontaneous colonic perforation (SCP) in young adults is extremely rare. In the absence of colonic disease and with clinical manifestations of connective tissue disorders, a genetic investigation for vEDS should be conducted, even in individuals without a family history. In patients with known vEDS, SCP may warrant more radical upfront surgical approaches such as total colectomy, as this syndrome frequently leads to new perforations. The treatment strategy must always consider the patient’s age, overall health, and preferences.

## References

[1] Kanaka S, Yamada T, Matsuda A, et al. Surgical management of colonic perforation in a patient with vascular Ehlers-Danlos syndrome with no family history: a case report. J Anus Rectum Colon 2020;4:201–5. 10.23922/jarc.2020-029 33134602 PMC7595682

[2] El Masri H, Loong TH, Meurette G, et al. Bowel perforation in type IV vascular Ehlers–Danlos syndrome. A systematic review. Tech Coloproctology 2018;22:333–41. 10.1007/s10151-018-1783-4 29700641

[3] Elhattabi K, Benghait H, Elbakouri A, et al. Total colectomy in vascular Ehlers Danlos syndrome a case report and literature review. Ann Med Surg 2021;71:102948. 10.1016/j.amsu.2021.102948 PMC8577335 34777791

[4] Augustin G, Radin I, Bubalo T, et al. Spontaneous sigmoid colon perforation and ruptured Subserosal (“zebra” pattern) small-bowel hematomas in type IV Ehlers–Danlos syndrome: a case report and a short review. J Clin Med 2024;13:4093. 10.3390/jcm13144093 39064133 PMC11278160

[5] Horino T, Miyamoto Y, Ohuchi M, et al. Repeated intestinal perforations in vascular Ehlers-Danlos syndrome: a case report of a novel mutation in the COL3A1 gene. Surg Case Rep 2023;9:78. 10.1186/s40792-023-01643-6 37171638 PMC10182206

[6] Funaki K, Akagi T, Shiroshita H, et al. A case of sigmoid colon perforation due to segmental absence of intestinal musculature (SAIM) accompanied by vascular Ehlers–Danlos syndrome: a case report. Surg Case Rep 2023;9:138. 10.1186/s40792-023-01721-9 37530898 PMC10397162

